# Integrated RNA and DNA sequencing improves mutation detection in low purity tumors

**DOI:** 10.1093/nar/gku489

**Published:** 2014-06-26

**Authors:** Matthew D. Wilkerson, Christopher R. Cabanski, Wei Sun, Katherine A. Hoadley, Vonn Walter, Lisle E. Mose, Melissa A. Troester, Peter S. Hammerman, Joel S. Parker, Charles M. Perou, D. Neil Hayes

**Affiliations:** 1 Lineberger Comprehensive Cancer Center, University of North Carolina at Chapel Hill, Chapel Hill, NC 27599, USA; 2 Department of Genetics, University of North Carolina at Chapel Hill, Chapel Hill, NC 27599, USA; 3 The Genome Institute at Washington University, St. Louis, MO 63108, USA; 4 Department of Biostatistics, University of North Carolina at Chapel Hill, Chapel Hill, NC 27599, USA; 5 Department of Epidemiology, University of North Carolina at Chapel Hill, Chapel Hill, NC 27599, USA; 6 Department of Medical Oncology, Dana-Farber Cancer Institute, Boston, MA 02215, USA; 7 Broad Institute of Harvard and MIT, Cambridge, MA 02142, USA; 8 Department of Internal Medicine, Division of Medical Oncology, Multidisciplinary Thoracic Oncology Program, University of North Carolina at Chapel Hill, Chapel Hill, NC 27599, USA

## Abstract

Identifying somatic mutations is critical for cancer genome characterization and for prioritizing patient treatment. DNA whole exome sequencing (DNA-WES) is currently the most popular technology; however, this yields low sensitivity in low purity tumors. RNA sequencing (RNA-seq) covers the expressed exome with depth proportional to expression. We hypothesized that integrating DNA-WES and RNA-seq would enable superior mutation detection versus DNA-WES alone. We developed a first-of-its-kind method, called *UNCeqR*, that detects somatic mutations by integrating patient-matched RNA-seq and DNA-WES. In simulation, the integrated DNA and RNA model outperformed the DNA-WES only model. Validation by patient-matched whole genome sequencing demonstrated superior performance of the integrated model over DNA-WES only models, including a published method and published mutation profiles. Genome-wide mutational analysis of breast and lung cancer cohorts (*n* = 871) revealed remarkable tumor genomics properties. Low purity tumors experienced the largest gains in mutation detection by integrating RNA-seq and DNA-WES. RNA provided greater mutation signal than DNA in expressed mutations. Compared to earlier studies on this cohort, *UNCeqR* increased mutation rates of driver and therapeutically targeted genes (e.g. *PIK3CA*, *ERBB2* and *FGFR2*). In summary, integrating RNA-seq with DNA-WES increases mutation detection performance, especially for low purity tumors.

## INTRODUCTION

Somatically acquired sequence mutations (nucleotide substitutions, insertions and deletions) fuel the initiation and progression of cancer (1). Knowledge of mutations in patient specimens informs therapeutic management (2,3), and in large patient cohorts, provides the basis to assess recurrently altered genes that may drive molecular pathogenesis (1,4–5). DNA whole exome sequencing (DNA-WES) is currently the popular technology to sequence cancer genomes and has led to an abundance of discoveries in many cancer types (4,6–8). However, detecting somatic mutations by DNA-WES with high sensitivity and specificity remains a challenge (7,9–10), as evidenced by validation rates of 73% in repeated sequencing and by large inter-rater disagreement among different groups analyzing the same sequencing data (7,10). The biggest challenge is high quality mutation detection in low purity tumors (2,9,11), which are prevalent in widespread cancer types such as breast and lung (12). Advances in somatic mutation detection could improve cancer genome characterization and lead to new diagnostic and therapeutic targets.

Somatic mutation detection is dependent on tumor features, the sequencing technology, and the method of statistical modeling (8–9,13–17). To detect somatic mutations, algorithms compare tumor and patient-matched germline sequencing based on a variety of models (4,6–7,9,13–17). A tumor's degree of normal contamination and clonal heterogeneity decrease tumor purity. Low purity affects the fraction of mutated DNA observed out of all DNA at a genomic site, the mutant allele fraction (MAF) (8,12). MAF is not often 100%, can be slightly above zero in low purity tumors, and varies across the genome depending on the prevalence of clones possessing a given mutation and on copy number alterations (7,9,12). DNA-WES targets roughly 200 000 exonic regions and, in practice, can yield depths of 100X or greater over targeted regions (4,6). DNA-WES has limitations including variable capture-efficiency and incomplete exome coverage (7,18). In cases of high MAF, mutation detection is straightforward as only a small number of reads are needed to detect the mutation with confidence. The combination of low depth and low MAF make mutation detection very difficult because of low statistical power, a result of the scant sample size in which to observe and detect the low prevalence mutation.

Increased mutation detection sensitivity and specificity could be achieved by statistical improvements, by increasing sequencing quantity or by increasing sequencing quality. In cancer profiling projects such as The Cancer Genome Atlas (TCGA) (4,6) and in clinical sequencing (2,19), DNA-WES is utilized for mutation detection while RNA sequencing (RNA-seq) (20) is performed for gene expression, fusion transcript and splicing analyses. Beyond those applications, RNA-seq provides an observation of the underlying tumor DNA sequence, via transcription, and can be used to detect sequence variants (21). In fact, we have previously used RNA-seq to confirm mutations from DNA-WES (4). A few earlier studies have used RNA-seq alone for genome-wide identification of somatic mutations (22–25) and germline variants (26,27). However, RNA-seq has challenges including dependency on gene expression, which limits the genes that can be measured for sequence mutations, and quality control requirements, which when not considered result in abundant false positive variants (11,21,28–30). For these reasons, RNA-seq has not been the standard for somatic mutation detection.

Herein, we posed the original hypothesis that integrating patient-matched tumor RNA-seq and tumor DNA-WES would enable superior mutation detection versus DNA-WES alone. We developed a first-of-its-kind method, *UNCeqR*, that simultaneously analyzes DNA-WES and patient-matched RNA-seq to detect somatic mutations genome-wide. *UNCeqR* was applied to large breast and lung cancer cohorts and evaluated with respect to simulation and whole genome sequencing validation. Subsequently, genome-wide analysis of *UNCeqR* mutations led to novel discoveries in tumor genomics.

## MATERIALS AND METHODS

### Data sources

DNA-WES and RNA-seq alignments in BAM (31) format for 176 lung squamous cell carcinoma cases and for 695 breast cancer cases were acquired from TCGA at https://cghub.ucsc.edu (Supplementary Table S1). RNA-seq were paired 50 nt read from Illumina HiSeq, aligned by MapSplice (4,32). DNA-WES were paired 76–100 nt reads from Illumina Genome Analyzer, aligned by BWA (33). All lung and breast cancer cases had germline DNA-WES, tumor DNA-WES and tumor RNA-seq and were referred to as the triplet cohorts. A subset of 12 lung and 91 breast tumors also had germline RNA-seq available and were referred to as the quadruplet cohorts. DNA whole genome sequencing (DNA-WGS) was acquired from TCGA for tumors in this cohort (breast: *n* = 43, lung: *n* = 17), which consisted of BWA alignments of paired 100 nt reads. Exonic coordinates were extracted from the TCGA Genome Annotation File (http://tcga-data.nci.nih.gov/docs/GAF/GAF.hg19.June2011.bundle/outputs/TCGA.hg19.June2011.gaf) and padded with 10 flanking positions, for a total of 222 055 exons. Published mutations (lung: LUSC_Paper_v8.aggregated.tcga.somatic.maf, breast: genome.wustl.edu_BRCA.IlluminaGA_DNASeq.Level_2.5.1.0.somatic.maf), expression subtypes, DNA copy number calls and tumor purity calls (12) were obtained when available from TCGA. Numerical purity calls of 1 with an incongruent ‘Low purity’ categorical call were censored.

### Sequencing quality filtering

The high quality data filter applies to alignments and genomic positions, similar to earlier studies (9,14). High quality sequenced bases from tumor alignments had base quality ≥20 and occurred in a parent alignment with the following properties: mapping quality ≥ 20, sum of reference mismatches insertions and deletions ≤2, a proper pair orientation, not a marked duplicate or qc-failure, not within the terminal two bases, and the singular best alignment. All bases from germline alignments were accepted. High quality genomic positions were those with germline depth ≥10, tumor high quality depth ≥5 in RNA or DNA, no homopolymer > 4 on either side of the site, proportion of high quality bases ≥0.25 in RNA or DNA, and without an insertion or deletion event at 10% allele fraction within 50 positions in germline sequencing. The high quality data filter was applied prior to detecting to tumor variant alleles. The high quality variant filter passes DNA or RNA variant alleles without significant strand bias compared to germline alleles (chi-square *P* < 0.01), with at least one read on both strands for indel variants, with major variant allele prevalence (the proportion of major variant reads out of all variant reads) ≥0.75, and a MAD of distance to the end of its aligned read sequence ≥1.

### Somatic mutation detection

The *UNCeqR* algorithm detected somatic mutations within exons based on input of tumor and patient-matched germline sequence alignments. The algorithm applied the following steps to each genomic site within exons:

1. filter for high quality data;
2. identify germline alleles from germline reads that have at least 2% allele prevalence;
  - add population polymorphisms and mapping artifact alleles to germline alleles (see following section ‘Population polymorphisms and mapping artifacts’).
3. Using tumor sequences:
  - let *g* be the number of reads matching germline alleles,
  - determine most frequent allele, that does not match germline alleles,
  - let *k* be the number of reads with this major variant allele,
  - let *n* = *k* + *g.*
4. If major variant allele is insertion or deletion, re-align nearby indel alleles:
  - scan 20 neighboring sites to find site *s* with maximum *k* and same major variant allele,
  - if current site is not *s.*
    1. Move major variant read count from current site to *s* by incrementing *k* at *s* and decrementing *g* at *s* by current site's major variant read count.
    2. Continue to next site.
5. If high quality variant filter is passed, apply statistical test, otherwise *P* = 1 if k = 0, else P = NA..

A set of mutation detection models applied the algorithm with different inputs and statistical models. *UNCeqR_DNA_* takes tumor DNA-WES as input and models the corresponding read counts by a beta-binomial distribution. For a variant site with read count $k_{{\rm DNA}}$, the *P*-value to assess whether this variant allele is a somatic mutation was calculated by

\begin{equation*}
P_{{\rm DNA}} = 1 - \sum\limits_{i = 0}^{k - 1} {\left( {\begin{array}{*{20}c} {n_{{\rm DNA}} } \\ i \\ \end{array}} \right)} \frac{{B\left( {i + \alpha _{{\rm DNA}} ,n_{{\rm DNA}} - i + \beta _{{\rm DNA}} } \right)}}{{B(\alpha _{{\rm DNA}} ,\beta _{{\rm DNA}} )}},
\end{equation*}

where *B* is the beta function, and *α*_DNA_ and *β*_DNA_ are parameters of the null distribution where the variant allele is not a somatic mutation. Specifically, *α*_DNA_ and *β*_DNA_ are estimated using randomly sampled sites until 50 000 have passed the high quality data filter in both tumor DNA-WES and tumor RNA-seq. In real data analysis, these sampled sites may include real somatic mutations and thus the estimates of *α* and *β* are conservative, which may lead to conservative *P*-value estimates. However, based on mutation rates reported in prior studies (8 mutations per 1 000 000 sites (4)), less than one mutation is expected in these sampled sites, and thus our estimates of *α* and *β* would be good approximations of the estimates from a set of non-somatic mutation sites. The *UNCeqR_RNA_* model is identical to *UNCeqR_DNA_* substituting tumor RNA-seq for tumor DNA-WES. The *UNCeqR_META_* model combines *P*-values from *UNCeqR_DNA_* and *UNCeqR_RNA_* if RNA and DNA have the same major variant allele irrespective of filtering; otherwise the *UNCeqR_META_ P*-value is set to that of *UNCeqR_DNA_*. In effect, this condition precludes sites with only RNA variant evidence, that are suggestive of RNA-editing (34,35), from being called somatic mutations. *UNCeqR_META_* combines *P*-values by the Stouffer method (36–38) with weights of the root of their sample size (read depth at the site) as follows:

\begin{eqnarray*}
&&P_{{\rm META}} = 1 - \\ &&\varPhi\left({\frac{{\varPhi ^{ - 1} \left( {1 - P_{{\rm DNA}} } \right)\sqrt {n_{{\rm DNA}} } + \varPhi ^{ - 1} \left( {1 - P_{{\rm RNA}} } \right)\sqrt {n_{{\rm RNA}} } }}{{\sqrt {n_{{\rm DNA}}^{} + n_{{\rm RNA}}^{} } }}} \right), \end{eqnarray*}

where $\varPhi$ is the standard normal cdf and $\varPhi ^{ - 1}$ is the inverse of $\varPhi$, i.e. the quantile function of the standard normal distribution. If the RNA major variant equals the DNA major variant and *P*_DNA_ = NA, *P*_META_ is set to *P*_RNA_. DNA and RNA variant read counts among putative false positives were unassociated supporting the usage of Stouffer's method (Supplementary Figure S1). Due to possible ambiguity around insertions and deletions (‘indels’) between DNA and RNA alignments, high quality variant sites with an insertion or deletion major variant allele in one alignment and with the same variant allele (insertion or deletion) occurring within 20 sites as the major variant allele in the other alignment were merged to have the same genomic position prior to statistical testing. This indel merge allowed indel variants sites between DNA and RNA that represent the same variant, to be recorded at the same site and allowed *UNCeqR_META_* to combine this DNA and RNA evidence despite slightly different representation in the sequence alignments. *UNCeqR* software consisted of modified samtools (31), Perl, R and VGAM (39). The total number of applied statistical tests is reported in *UNCeqR* output to provide interested users the possibility of multiple testing adjustment.

### Population polymorphisms and mapping artifacts

Population-level polymorphisms were acquired from dbSNP common version 137 via the UCSC genome browser (40). Variant alleles caused by ambiguous mapping artifacts were calculated by BlackOps (41) using 2 × 50 paired-end reads aligned by MapSplice. *UNCeqR* was applied to 45 TCGA RNA-seq of matched normal tissue specimens (not part of the lung or breast cohorts) to detect non-reference sequence variants, representing further germline polymorphic and alignment artifact alleles. These alleles always augmented germline genotype in *UNCeqR*, thus preventing somatic mutation detections with these alleles even if unobserved in a given germline sequencing.

### Mutation annotation and analysis

Sequence mutations were annotated with a gene, a predicted transcript and protein alteration using Annovar (version 8/23/13) (42) and RefSeq gene models. Non-silent mutations referred to non-silent substitution, insertion and deletion mutations within translated regions and splice-site mutations. MAFs were compared by one-sided Fisher's exact tests on mutant versus germline read counts with significant results having false discovery rate < 5%*.*  Sequence alignments were visualized using the Integrative Genomics Viewer (43).

### Germline variant analysis

Patient germline variants relative to the reference genome were detected in germline DNA-WES and patient-matched germline RNA-seq using *UNCeqR_META_* without population polymorphism or mapping artifact allele augmentation, *P* ≤ 1.1e−9. Germline variant allele fractions were defined and compared between DNA and RNA, using the procedure described for somatic mutations.

### Simulation analysis

A novel simulation strategy was followed (diagrammed in Supplementary Figure S2). Using chromosome 2, simulated tumor genomes were generated by randomly sampling 500 sites from exons to define positive mutation sites while the remainder of exon sites served as negative mutations. For the positive sites, mutant alleles (substitution, insertion or deletion) were randomly sampled at rates 90, 5 and 5%. For insertion and deletion alleles, allele lengths of 1–6 were randomly sampled at rates 60, 20, 9, 5, 5 and 1%. Positive mutations were spiked into germline DNA-WES and RNA-seq sequencing by editing a specified MAF of read alignments overlapping the site, producing simulated tumor alignments. ‘V’ characters were used for substitutions and insertions to avoid overlap with germline genotype. Simulated tumor alignments contained a subset of the total positive mutations because the alignment may have minimal or zero depth at some positive sites, reflecting reality that a sequencing technology does not cover every site in the genome at high depth and enabling simulated mutations to occur at RNA-seq and DNA-WES uniquely covered sites. Original tumor sequencing served as simulated germline sequencing. Simulated germline sequencing contained the original somatic mutations, which had the effects of expanding germline genotype with additional alleles and not triggering variant detection. *UNCeqR* models were applied to these simulated data. Limiting to sites with at least a germline depth of 10, model detections were compared to the truth to define receiver operating characteristic (ROC) curves (44). A pair of models was compared by their difference in area under the curve over the false positive rate range of 0 to 1 × 10^−5^. A *P*-value was defined using a distribution of differences in area under the curve calculated from 100 permuted models in which the rank of the discrimination threshold (i.e. *P*-value) between the models at each genomic site was randomly shuffled.

### Mutation detection by other programs

Strelka v2.0.8 (17) was executed on tumor and germline DNA-WES using recommended settings for BWA alignments (strelka_config_bwa_default.ini), DNA-WES (isSkipDepthFilter = 1) and filtering (passed). SNVMix2 (13) was executed upon RNA-seq using default settings.

### Validation analysis

Within exonic regions, true positive and false positive mutation detections were defined using patient-matched DNA-WGS alignments based on a published procedure for exome mutation validation (4). Tumor and germline DNA-WGS BAM files were downloaded from https://cghub.ucsc.edu. Specifically, tumor and germline DNA-WGS were interrogated at each predicted mutation using samtools (31) with no filtering. True positive mutation predictions met one of two conditions: (1) germline depth ≥ 10 and read count of predicted mutant allele ≥1 in tumor and zero in germline; or (2) germline depth ≥10, proportion of mutant allele in germline sequencing not significantly > 2% (proportions test, *P* > 0.25) and proportion of mutant allele in tumor significantly greater than in germline (proportions test, *P* < 0.05). Otherwise, false positive mutation predictions had germline DNA-WGS depth ≥10, and had depth in tumor DNA-WGS providing ≥80% power to detect the mutant allele based the predicted MAF. Power was estimated by a binomial distribution, a null probability of 3 × 10^−3^, an alpha of 0.05, the observed depth in DNA-WGS and an alternate probability of the predicted DNA MAF. The number of true positives and false positives were tabulated at each model discrimination threshold, i.e. *P*-value or score. The step function of these points (number of false positives versus number of true positives) generated a performance curve in absolute counts that is equivalent to a ROC curve without the denominators of total positives and negatives, which were constant and unknown for the validation cohort. Between models, performance curves were compared by area under the curve from 0 to 3000 false positives and by the number of true positives (proportional to sensitivity) at fixed numbers of false positives (proportional to 1 − specificities) of 250, 500 and 1000). *P*-values were calculated to provide evidence for the change in area under the curve and sensitivity estimates using permutation (see ‘Simulation analysis’ methods).

## RESULTS

### Mutation detection models

Existing methods to detect somatic mutations are based on either DNA sequencing alone or on RNA sequencing alone and do not integrate more than one type of sequencing (9,13–17). In order to test whether integrating DNA-WES and RNA-seq enables superior somatic mutation detection versus the current standard of DNA-WES alone, a new method was developed, called *UNCeqR*. *UNCeqR* contains different models for detecting somatic mutations based on different sequencing input and statistical modeling. Briefly, *UNCeqR_META_* integrates tumor DNA-WES and RNA-seq, *UNCeqR_DNA_* uses tumor DNA-WES, and *UNCeqR_RNA_* uses tumor RNA-seq. *UNCeqR* software is available at http://lbg.med.unc.edu/tools/unceqr.

### Evaluation in simulated tumor sequencing

To test our hypothesis that somatic mutation detection based on integrated RNA-seq and DNA-WES is superior to that based on DNA-WES alone, simulated tumor genomes were generated so that the entire genome space is a completely defined truth of positive and negative somatic mutations. In brief, for each patient's sequencing, 500 mutant sites were sampled, for each site a mutant allele was randomly sampled, and then aligned reads in the real RNA-seq and DNA-WES were edited to have the mutant allele at a rate of a fixed MAF (Supplementary Figure S2). By using real sequencing as the basis of the simulation, authentic sequencing depths, random errors (sequencing and alignment) and patients’ germline variants were preserved.

**Figure 1.**
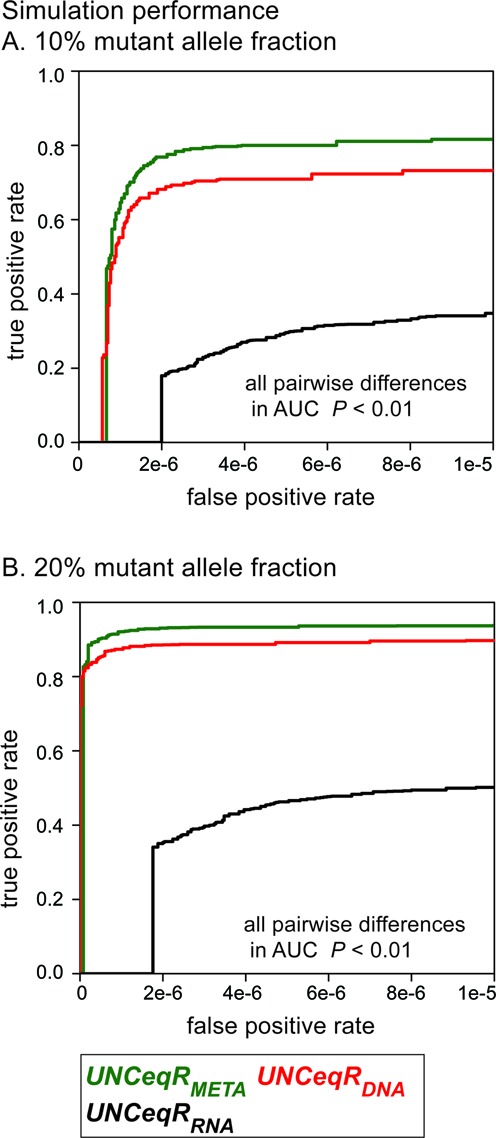
Mutation detection performance in simulated tumor genomes. Model performance is displayed as receiver operating characteristic curves. Sensitivity plateaus below 1 because simulated mutations include sites with zero tumor sequencing depth in DNA and/or RNA (see ‘Simulation analysis’ methods).

Sequencing from the lung cancer quadruplet cohort was used for simulation. Patients’ DNA-WES and RNA-seq had large and similar numbers of sequenced nucleotides (DNA-WES median: 10.6 billion, RNA-seq median: 10.2 billion; Kruskal-Wallis *P* = 0.54) indicating no significant imbalance in total sequencing. *UNCeqR* models were applied to the simulated tumor sequencing and detected mutations were compared against the truth by receiver operating characteristic curves. In simulations with a 10% MAF (Figure 1A), the *UNCeqR_META_* model had significantly superior performance over *UNCeqR_DNA_* (difference in area under the curve, *P* < 0.01); in other words, *UNCeqR_META_* achieved a greater true positive rate (greater sensitivity) at the same false positive rate (same specificity) than *UNCeqR_DNA_*. In simulations with a 20% MAF (Figure 1B), *UNCeqR_META_* continued to be superior to *UNCeqR_DNA_* (difference in area under the curve, *P* < 0.01) although the gain in 20% MAF simulations was less (roughly 50% less) than the gain in 10% MAF simulations. This demonstrates that adding RNA-seq improved sensitivity, particularly when the mutation signal, that is MAF, was low. *UNCeqR_META_* and *UNCeqR_DNA_* had large and clear superior performance to *UNCeqR_RNA_*, which incurred false positives at a higher rate. Alternative ways to integrate RNA and DNA (taking the union or intersection of *UNCeqR_DNA_* and *UNCeqR_RNA_*) were both inferior to *UNCeqR_META_* (Supplementary Figure S3). Therefore, in simulation, *UNCeqR_META_* achieved superior performance over *UNCeqR_DNA_*, with the largest gains occurring in mutations with low MAF.

### Validation by whole genome sequencing

**Figure 2.**
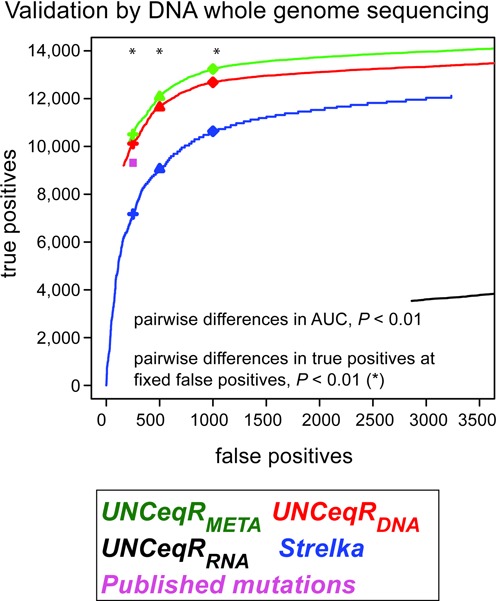
Validation of mutation detection by whole genome sequencing. The number of true positives and false positives of mutation detection models are plotted as step functions. At fixed false positive totals (250, 500 or 1000), each pair of models was compared for differences in number of true positives (*). The published mutation set (4,6) did not include mutation rankings and was not amenable to rank-based statistical analysis.

To validate the superior performance of integrated DNA-WES and RNA-seq mutation detection (*UNCeqR_META_*) over DNA-WES only detection (*UNCeqR_DNA_*), tumor and germline whole genome DNA sequencing (DNA-WGS) was used as an independent measure of truth for evaluating DNA-WES and RNA-seq mutation detections. Following a published validation procedure (4), mutation detections were interrogated in patient-matched DNA-WGS to determine if a mutation detection was a true positive, that is present in the tumor specimen and absent from the germline specimen, or false positive, that is absent from the tumor specimen or present in the germline specimen. For each mutation model, true positives and false positives were summed at each discrimination threshold (e.g. *P*-value) to generate a performance curve by which true positive rates could be compared at the same false positive rates (see methods for further description). These curves demonstrated that *UNCeqR_META_* achieved overall superior performance than *UNCeqR_DNA_* (difference in area under the curve, *P* < 0.01) and at fixed false positive thresholds (250, 500 and 1000), thus, validating the result from simulated tumor genomes (Figure 2). Therefore, in real tumor sequencing, integrated DNA and RNA mutation detection by *UNCeqR_META_* outperformed DNA-only mutation detection.

Other models displayed overall reduced performance relative to *UNCeqR_META_* and *UNCeqR_DNA_*. As another DNA-only control, a leading (45) DNA-WES mutation caller from Illumina, *Strelka* (17), was run on the same DNA-WES. *Strelka* exhibited inferior performance overall, smaller true positive rates at fixed false positive rates, and never achieved the sensitivity of *UNCeqR_META_* or *UNCeqR_DNA_* (Figure 2). Strelka had greater sensitivity than *UNCeqR_META_* or *UNCeqR_DNA_* at the highest extreme of specificity; however, at *UNCeqR*'s minimum false positive rate, *Strelka*'s sensitivity was only ∼70% of either *UNCeqR* model. Providing another DNA-only control, previously published mutations of this cohort made by heterogeneous pipelines (4,6,9,15–16) had reduced sensitivity than *UNCeqR_META_* and *UNCeqR_DNA_* at the same false positive rate (256 false positives). At this false positive rate, indel mutation detections were rare in all models (maximum 1.7%) with *UNCeqR_META_* and *UNCeqR_DNA_* having no significant difference in indel precision (number of true positives divided by the sum of false positives and true positives, 92 and 96%, respectively) but both having greater indel precision than *Strelka* (83%) and previously published mutations (82%) (proportions test, *P* < 0.001). Taking the union or intersection of *UNCeqR_DNA_* and *UNCeqR_RNA_* had higher false positive rates and inferior performance than *UNCeqR_META_* or *UNCeqR_DNA_* (Supplementary Figure S4A). Integrating Strelka with an RNA-seq mutation detector, SNVmix, did not result in superior performance versus Strelka, *UNCeqR_DNA_* or *UNCeqR_META_* (Supplementary Figure S4A). Providing a separate source of validation, *UNCeqR_META_* detected nearly all mutations that were published as validated by targeted resequencing within this cohort (up to 97%, depending on the model threshold; Supplementary Figure S5). Repeating this analysis with a slightly increased true positivity definition, minimum two confirming tumor WGS DNA reads, maintained all findings listed above (Supplementary Figure S4B).

### Increased mutation signal in RNA-seq

**Figure 3.**
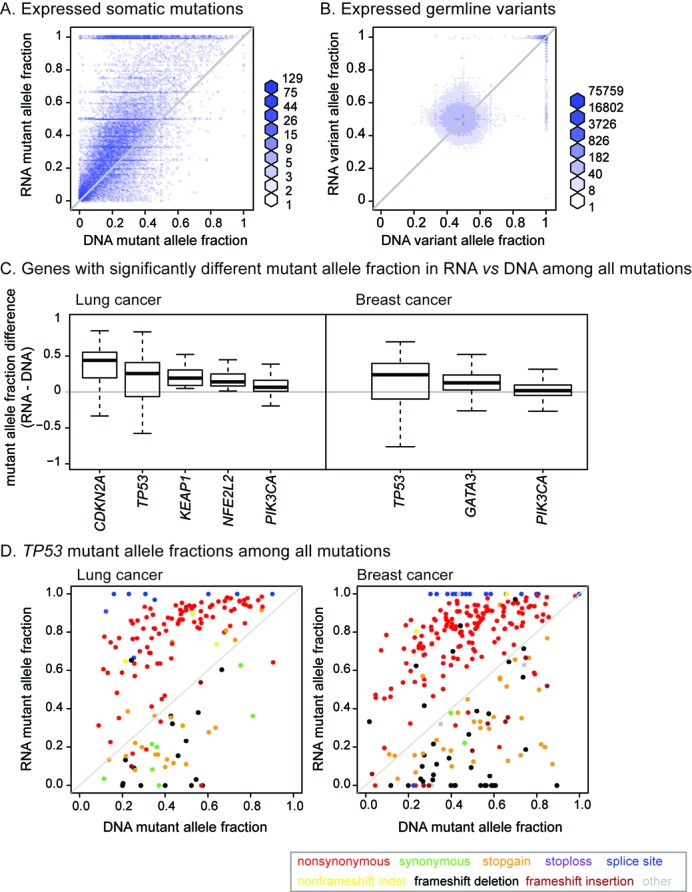
Mutation signal in RNA versus DNA. Mutant allele fraction distributions of *UNCeqR_META_* expressed mutations from the lung triplet cohort tumor sequencing (**A**). Germline variant allele fraction distributions of expressed germline variants from lung quadruplet cohort germline sequencing (**B**). Diagonal lines indicate equal allelic fraction between DNA and RNA, with points above the diagonal having greater allelic fraction in RNA, below the diagonal greater allelic fraction in DNA. Breast cancer somatic mutation and germline allele distributions in Supplementary Figure S6. Distributions of MAF difference among driver genes having a significant difference in MAF over all mutations (**C**). MAF distributions for all *TP53 UNCeqR_META_* mutations, expressed and unexpressed (C and **D**).

To analyze integrated mutation detection across larger cohorts, *UNCeqR* was applied to the lung and breast triplet cohorts (*n* = 871) and using model thresholds with the same empirically estimated specificity (500 false positives in DNA-WGS validation sequencing, marked as triangle point in Figure 2, *UNCeqR_META_ P*-value ≤ 1.1 × 10^−9^, *UNCeqR_DNA_ P*-value ≤ 9.3 × 10^−9^). About half (49%) of *UNCeqR_META_* mutations had no RNA evidence and were based only on DNA evidence. Surprisingly among *UNCeqR_META_* expressed somatic mutations (those with RNA and DNA mutant read evidence), the MAF in RNA was often significantly greater than in DNA (lung: 21% of expressed mutations, breast: 17%, fdr < 0.05) (Figure 3A and Supplementary Figure S6A). This increase was often >2-fold (lung: 12% of expressed mutations, breast: 11%). In contrast, DNA MAF was significantly greater than RNA MAF at much lower frequency (lung: 2% of expressed mutations, breast: 3%, fdr < 0.05). As a control, germline variants were detected in germline DNA-WES and patient-matched germline RNA-seq relative to the reference genome by *UNCeqR_META_* under the same settings as somatic mutation detection (Figure 3B and Supplementary Figure S6B). In contrast to expressed somatic mutations, expressed germline variants displayed rare significant differences in allele fraction (RNA greater than DNA: lung: 0.8%, breast: 0.7%; DNA > RNA: lung 0.1%, breast: 0.3%). Therefore, the prevalent, increased mutation signal in RNA-seq was cancer-specific.

In addition to the genome-wide phenomenon, the increased mutation signal in RNA versus DNA might additionally be frequent in cancer driver genes. Lung and breast cancer's driver genes (4,6) with at least 10% prevalence were analyzed for differences in RNA to DNA MAF across all mutations, whether expressed or not. Eight driver genes had significantly different MAF between DNA and RNA (Wilcoxon signed rank test, fdr < 0.05; Figure 3C). All of these genes had greater median MAF in RNA than in DNA, including an oncogene, *PIK3CA* and tumor suppressors, such as *TP53*. The *TP53* MAF distributions of lung and breast cancer had remarkable similarities (Figure 3D), in that nonsynonymous and splice site mutations had extremely high RNA MAF relative to DNA MAF, often 2-fold greater. Stop-gain and frameshift mutations in *TP53* had greater MAF in DNA versus RNA but these decreases were less common and had a smaller magnitude in MAF difference. The *TP53* results extend an earlier report in lung cancer using direct sequencing of *TP53* RNA transcripts which found mutant transcript predominant expression (46). In summary, expressed mutations tend to have larger mutation signal in RNA than in DNA. Importantly, this effect was common among driver genes, suggesting that integrating DNA and RNA for mutation detection provides the best opportunity to identify cancer causing mutations.

Because DNA copy number can affect the quantity of tumor versus germline DNA at a locus, tumor DNA copy number alterations were compared among mutations with a significantly greater MAF in RNA versus DNA and *vice versa*. Mutations with greater MAF in RNA exhibited a small (roughly 5%) relative increase in DNA copy number deletions (Supplementary Figure S7), suggesting that RNA is beneficial to detect mutations in regions of genome deletion. MAF differences in *TP53* mutations did not associate with either DNA amplifications or DNA deletions (Supplementary Figure S7).

### Large gains in low purity tumors

**Figure 4.**
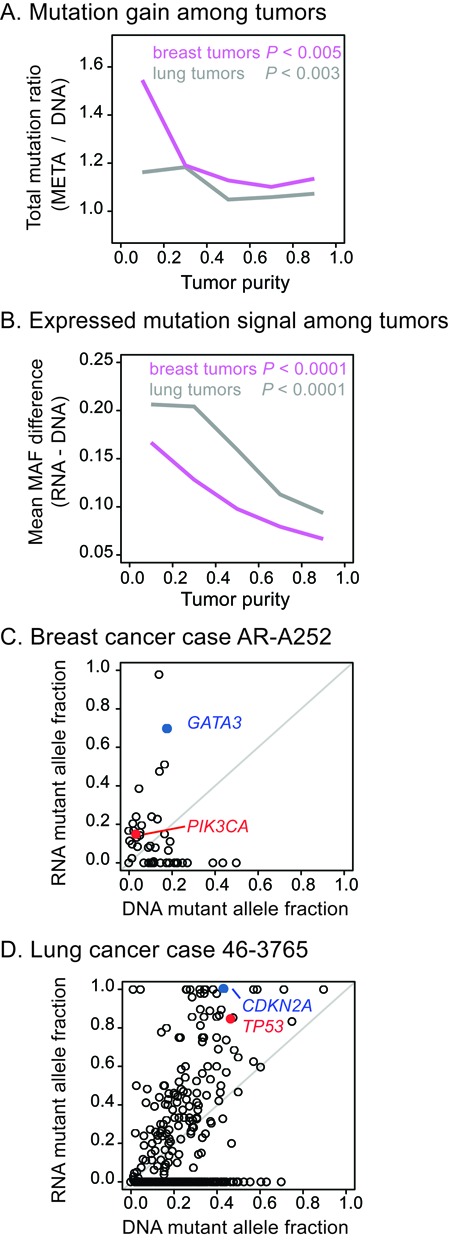
Tumor purity effects on mutation detection. Lines summarize breast and lung triplet cohorts, displaying total mutation ratios (**A**) or mean mutant allele fraction difference within expressed mutations (**B**) among tumors, binned by tumor purity quintile and plotted at midpoint. Pearson's correlation tests compared the association of mutation ratio and MAF associations among triplet cohort tumors (*P*). MAF distributions from two exemplar low purity tumors’ mutations (**C** and **D**). Diagonal lines indicate equal MAF in DNA-WES and RNA-seq, with mutations above the diagonal having greater MAF in RNA, below the diagonal greater MAF in DNA. Unexpressed mutations are marked along the horizontal axes in (C and D).

Because low tumor purity (caused by normal contamination and multiple clones) can affect mutation detection (2,8), the outcome of integrating RNA-seq and DNA-WES in mutation detection was compared among tumors by their purity. The rate of mutation gain after adding RNA-seq to DNA-WES was non-uniform both in the breast and lung triplet cohorts, such that the greatest gains occurred in tumors having the lowest purity. Specifically, tumors’ total mutation ratio (the number of mutations detected by *UNCeqR_META_* over *UNCeqR_DNA_*) had significant negative correlation with tumor purity in both lung and breast cancer (Figure 4A). Mutation gains were largest among tumors with purity <40%. In addition, tumors’ average difference in mutation signal between RNA and DNA (the mean difference of RNA MAF to DNA MAF across all expressed *UNCeqR_META_* mutations) also had significant negative correlation with tumor purity both in lung and breast cancer (Figure 4B). Therefore, tumors with low purity had the largest RNA-seq mutation signal and gained the most new mutations after incorporation of RNA-seq evidence.

**Figure 5.**
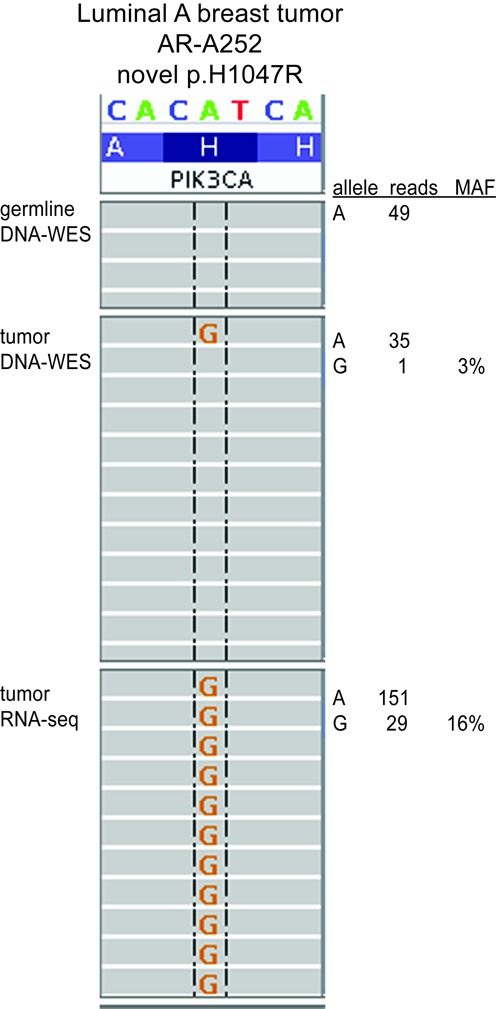
Example of somatic mutation only detectable by RNA and DNA integration.  Mutation detected by *UNCeqR_META_P* = 1e-16. Read alignment display from integrative genomics viewer (43) for a low purity breast tumor at the major mutational hotspot of *PIK3CA* (47).

Examples of low purity tumors with large mutation gains include a low purity breast tumor that had 1.8 total mutation ratio and a mean 0.18 difference in mutation signal among expressed mutations. Two of this tumor's mutations with much larger signal in RNA than DNA occurred in *PIK3CA* (p.H1047R) and *GATA3* (p.S412fs) (Figure 4C). These mutations occur in major mutational hotspots (47) and are also characteristic molecular drivers for the Luminal A expression subtype (6,48) of which this tumor is a member. Incorporation of RNA-seq evidence was essential to identify these two driving mutations; e.g. there was only 1 DNA read with the *PIK3CA* mutation but 29 mutant reads in RNA-seq (Figure 5). An example lung tumor had a 1.2 total mutation ratio and an average 0.22 difference in mutation signal among expressed mutations including *CDKN2A* (p.H98P) and *TP53* (p.R273H) which exhibited very large RNA MAF (at 100 and 84%) relative to DNA MAF (at 43 and 46%) (Figure 4D). These *PIK3CA*, *GATA3* and *TP53* mutations were not detected by earlier studies utilizing DNA-WES alone (4,6), emphasizing the advantage of RNA integration. In summary, the addition of RNA-seq to DNA-WES substantially boosted mutation sensitivity for low purity tumors.

### Increased mutation rates of driver and therapeutically-targeted genes

**Figure 6.**
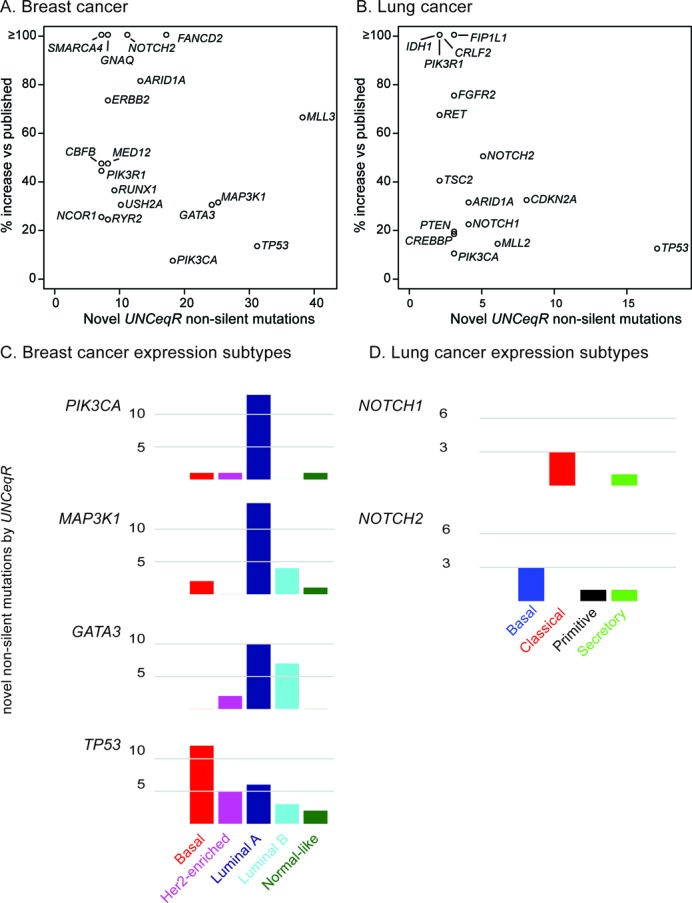
Novel mutation discoveries in cancer-relevant genes. Increases in mutation absolute count versus relative increase are displayed for selected genes (**A** and **B**). Percentage increase is the number of novel *UNCeqR_META_* mutations over the number of published mutations (4,6)for a gene. Absolute counts for select genes among breast (**C**) and lung (**D**) cancer expression subtypes.

To determine if *UNCeqR_META_* made new mutation discoveries in patients’ tumor genomes, *UNCeqR_META_* mutations were compared to previously published patient mutation profiles on the triplet cohorts (4,6). Specifically, tumors’ non-silent mutations (those that change protein sequence and can contribute to cancer development) of *UNCeqR_META_* that were novel compared to published profiles were tabulated within genes known to be relevant in cancer development (187 genes, from the Cancer Gene Census (49) and published driver genes (4,6)). Five hundred and sixty-seven novel mutations were detected covering 67% of these cancer-relevant genes. 69% of these novel mutations had DNA-WES and RNA-seq evidence, indicating that the addition of RNA contributed to the vast majority of these novel mutations. Grouped by patients, 44% of patients’ tumors had an increase of at least one new mutation in this cancer-relevant gene set, and among patient tumors with zero published mutations in this gene set, 42% had at least one new mutation discovered by *UNCeqR_META_*. Grouped by gene, many of these novel mutations comprised large gains in absolute counts and in percent increase (Figure 6A and B), including *MAP3K1* and *GATA3* in breast cancer, and *NOTCH2* and *CDKN2A* in lung cancer. These gains spanned all nucleotide mutation types (substitution, insertion and deletion) and protein coding impacts; for instance, novel *GATA3* mutations had abundant novel frameshift insertion, frameshift deletion, non-synonymous and nonsense mutations (Supplementary Figure S8). Notably, mutation rates for genes targeted by drugs were increased by *UNCeqR_META_*, specifically, *PIK3CA*, *FGFR2* and *ERBB2.* Therefore, *UNCeqR_META_* largely advanced published, state-of-the-art mutation profiles with cancer-relevant mutations by utilizing the integration of RNA-seq and DNA-WES.

Breast cancer subtypes (48) were previously found to have distinct rates of mutations across four genes (*TP53*, *GATA3*, *MAP3K1* and *PIK3CA*) and, in combination with other evidence such as pathway alterations, are understood to be driven by their distinct somatic alterations (6). Across these four genes, novel mutations detected by *UNCeqR_META_* occurred most frequently in tumors of the same expression subtype as had been previously reported. Specifically, the greatest number of novel mutations occurred in the following subtypes: *TP53* in Basal, *MAP3K1* in Luminal A, *PIK3CA* in Luminal A and *GATA3* in Luminal A and Luminal B (Figure 6C). In lung cancer, there were appreciable increases in *NOTCH1* and *NOTCH2*. The largest numbers of novel *UNCeqR_META_ NOTCH1* and *NOTCH2* mutations occurred in different lung cancer expression subtypes (50) of Classical and Basal, respectively (Figure 6D). Combining novel *UNCeqR* non-silent mutations with those previously reported, both of these genes now had significant association with expression subtype (*NOTCH1* Fisher's test *P* < 0.02; *NOTCH2* Fisher's test *P* < 0.03). Therefore, the advance of *UNCeqR_META_* over published mutation profiles included new subtype-specific driving mutations, new putative subtype-specific driver genes, and new patients with mutations in driver genes.

## DISCUSSION

Herein, we sought to determine if adding patient-matched RNA-seq to DNA-WES would improve somatic mutation detection. To this end, we developed *UNCeqR*, a first-of-its-kind method, that integrates RNA-seq and DNA-WES to detect somatic mutations. By simulation and validation in whole genome sequencing, the *UNCeqR_META_* model that integrates DNA and RNA had significantly superior performance to models based on DNA alone (*UNCeqR_DNA_*, Strelka and published mutation profiles). Then, we applied *UNCeqR* to large breast and lung cohorts (*n* = 871) and analyzed their integrated RNA and DNA mutations, resulting in several novel characterizations of tumor genomics.

We report for the first time a remarkable finding that low purity tumors experience the largest gains in total mutations and in mutation signal (MAF) when adding RNA-seq to DNA-WES. Also, we originally report that that MAF tends to be elevated in RNA versus DNA among expressed genes, and that this phenomenon is cancer-specific. Based on these observations, we conclude that rare cancerous cells within a tumor may exhibit over-expression relative to the tumor's normal cells, which increases the concentration of cancer cell's mutations in a locus’ expressed transcripts, thus boosting the RNA mutation signal. In contrast, low purity tumors’ DNA mutation signal, even if copy number altered, may be drowned out by the normal cell DNA and cannot achieve the magnitude of the RNA mutation signal. High purity tumors’ smaller increases in RNA mutant allele signal versus DNA could be caused by mutant allele-specific expression or the presence of minor cancer clones within the tumor. In summary, RNA-seq when added to DNA-WES is particularly useful for mutation detection in low purity tumors.

For mutations with therapeutic significance, highly sensitive and specific assays are essential for informing patient therapy and for clinical trials investigating new agents. Relative to published mutations derived from DNA-WES alone, the *UNCeqR_META_* mutations, derived from patient-matched DNA-WES and RNA-seq, increased the numbers of patients with mutations in genes that are targets for several drugs in clinical trials, such as *PIK3CA*, and *ERBB2*, and for drugs with correlative evidence, such as *FGFR2* (51). Clinical trials such as NCT01670877 which involve *ERBB2* sequencing (52) may be influenced to include RNA-seq due the large mutation rate increase reported here. Although the relative increase in *PIK3CA* mutations was modest compared to other genes in breast cancer, this improved sensitivity is vital for affected patients and could lead to positive clinical trial outcomes. For example, some novel canonical mutations in *PIK3CA* had many mutant reads in RNA-seq but only a few mutant reads in DNA-WES, such as the example Luminal A tumor with a single DNA mutant read in the *PIK3CA* hotspot. This study's results support that RNA sequencing could be beneficial when added to DNA sequencing in clinical settings.

Future studies could explore alternative ways to integrate DNA and RNA sequencing, beyond *UNCeqR_META_*, which is the first method of this kind. *UNCeqR_META_* applied the same quality filters for DNA and RNA, and potentially different filters could be beneficial. *UNCeqR_META_* includes a basic indel realignment, and integrated DNA and RNA reassembly could potentially be beneficial. Different statistical modeling could further advance the performance displayed by *UNCeqR_META_* over DNA only based methods. Balancing sensitivity and specificity is important in applying and developing mutation detectors. Receiver operating characteristic curve analysis, such as that presented in this study, enables assessment of sensitivity and specificity tradeoffs between alternate models.

Integrated RNA-seq and DNA-WES mutation detection is important because it boosts sensitivity in low purity tumors, in therapeutically-relevant genes and in driver genes, relative to DNA-only detection. Integrated mutation detection could also enable more inclusive cohort profiling studies that censor tumors based on purity and could lead to more comprehensive characterizations of cancer genomes. In conclusion, integrating DNA-WES and RNA-seq by *UNCeqR_META_* increases mutation detection performance and was extremely beneficial for low purity tumors.

## AVAILABILITY

*UNCeqR* software is available at http://lbg.med.unc.edu/tools/unceqr. *UNCeqR* mutation detections for the lung and breast cohorts are available at https://tcga-data-secure.nci.nih.gov/tcgafiles/tcga4yeo/tumor/ and http://lbg.med.unc.edu/tools/unceqr.

## SUPPLEMENTARY DATA

Supplementary Data are available at NAR Online.

SUPPLEMENTARY DATA
