# Supplementary material for: Integrated RNA and DNA sequencing improves mutation detection in low purity tumors
Source: Nucleic Acids Res. 2014 Jun 26;42(13):e107. doi: 10.1093/nar/gku489 (PMC4117748; doi:10.1093/nar/gku489)
Supplement: SUPPLEMENTARY DATA [file supp_42_13_e107__index.html]

Integrated RNA and DNA sequencing improves mutation detection in low purity tumors — SUPPLEMENTARY DATA 

# Integrated RNA and DNA sequencing improves mutation detection in low purity tumors

## SUPPLEMENTARY DATA

**Files in this Data Supplement:**

- Supplementary Data
- Supplementary Data
