# Supplementary material for: Integrated RNA and DNA sequencing improves mutation detection in low purity tumors
Source: Nucleic Acids Res. 2014 Jun 26;42(13):e107. doi: 10.1093/nar/gku489 (PMC4117748; doi:10.1093/nar/gku489)
Supplement: SUPPLEMENTARY DATA [file supp_gku489_1.pdf]

Supplementary material for:

Wilkerson MD et al (2014), Integrated RNA and DNA sequencing improves mutation detection in low purity tumors.

Supplemental Figures: 8

Supplemental Tables: 1

Figure S1: Variant counts among putative false positives.

Points mark variant read counts for putative false positive variant sites from germline lung quadruplet cohort sequence *vs* the reference by *UNCeqR*$_{DNA}$ and *UNCeqR*$_{RNA}$. Population polymorphisms and artifact sites were removed, and the remaining variant sites are consequences of alignment, sequencing, and unknown sources and are therefore putative false positive variants. Variant read counts between RNA-seq and DNA-WES at these sites are not positively associated (Spearman's correlation test, $P > 0.999$).

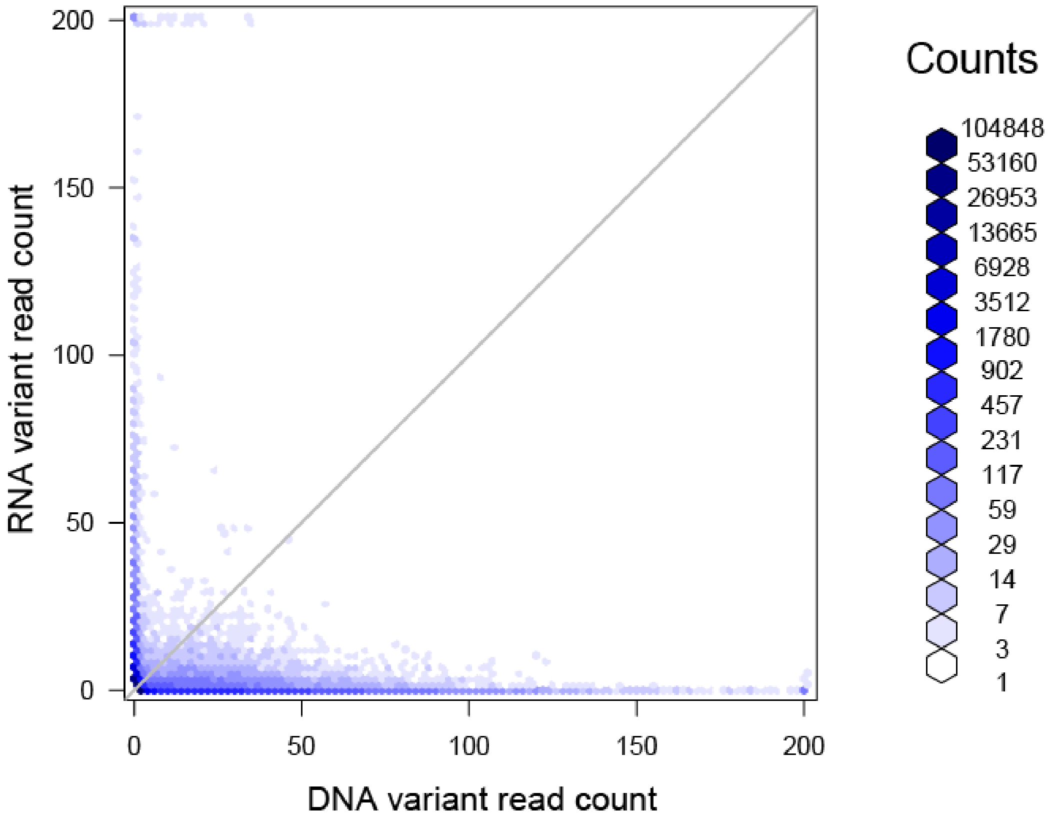

Fig S2: Simulation scheme.
Flow chart presents strategy for generating simulated tumor genomes and alignments and for applying *UNCeqR* methods on these simulated data.

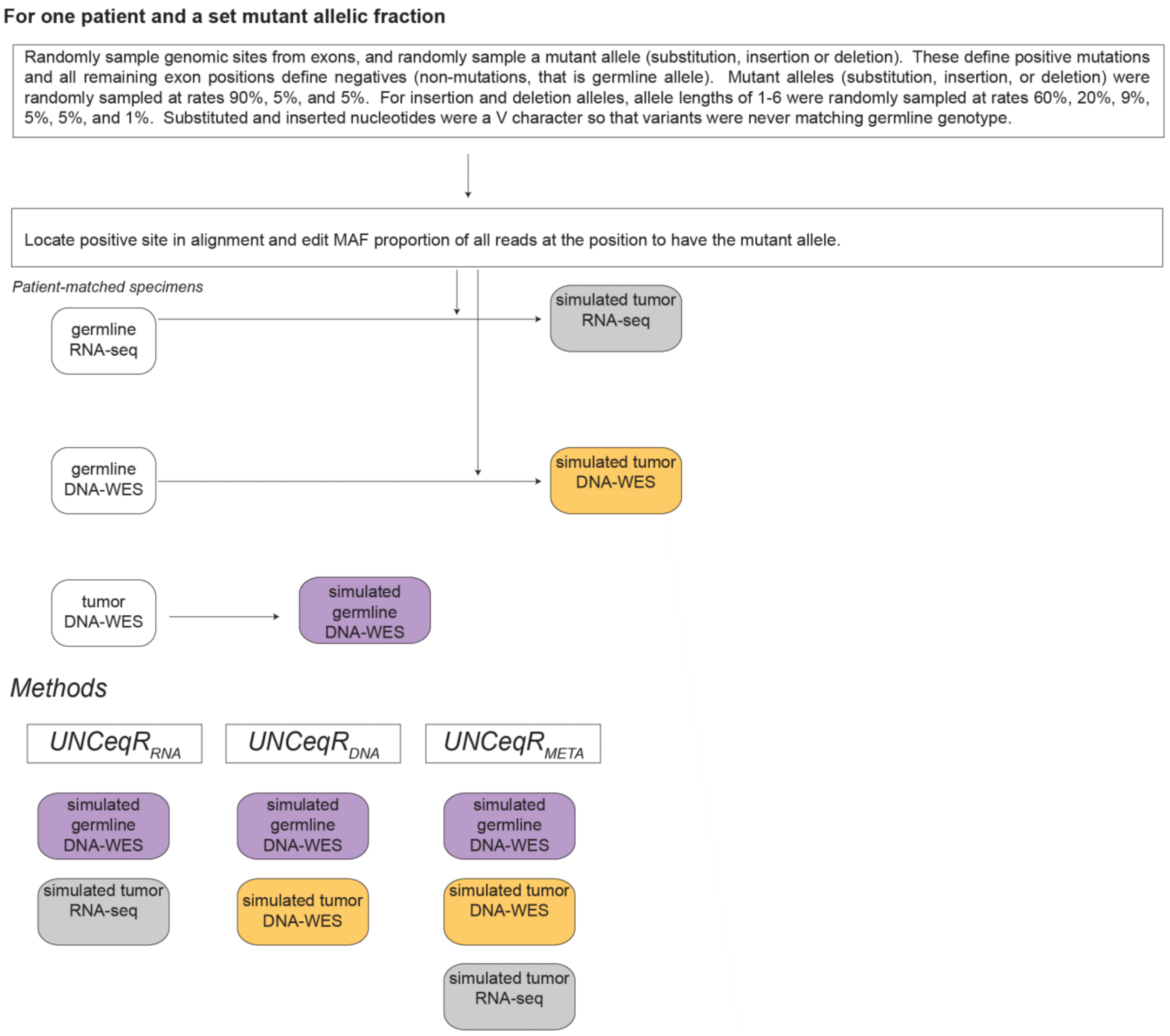

Figure S3: Extended performance in simulated tumor genomes.

Union refers to taking the union of mutation detections from $UNCeqR_{RNA}$ and $UNCeqR_{DNA}$, which was defined as the minimum p-value between the models. Intersection refers to taking the intersection of mutation detections from $UNCeqR_{RNA}$ and $UNCeqR_{DNA}$, which was defined as the maximum p-value between the models so that both models are making the detection at the p-value or less.

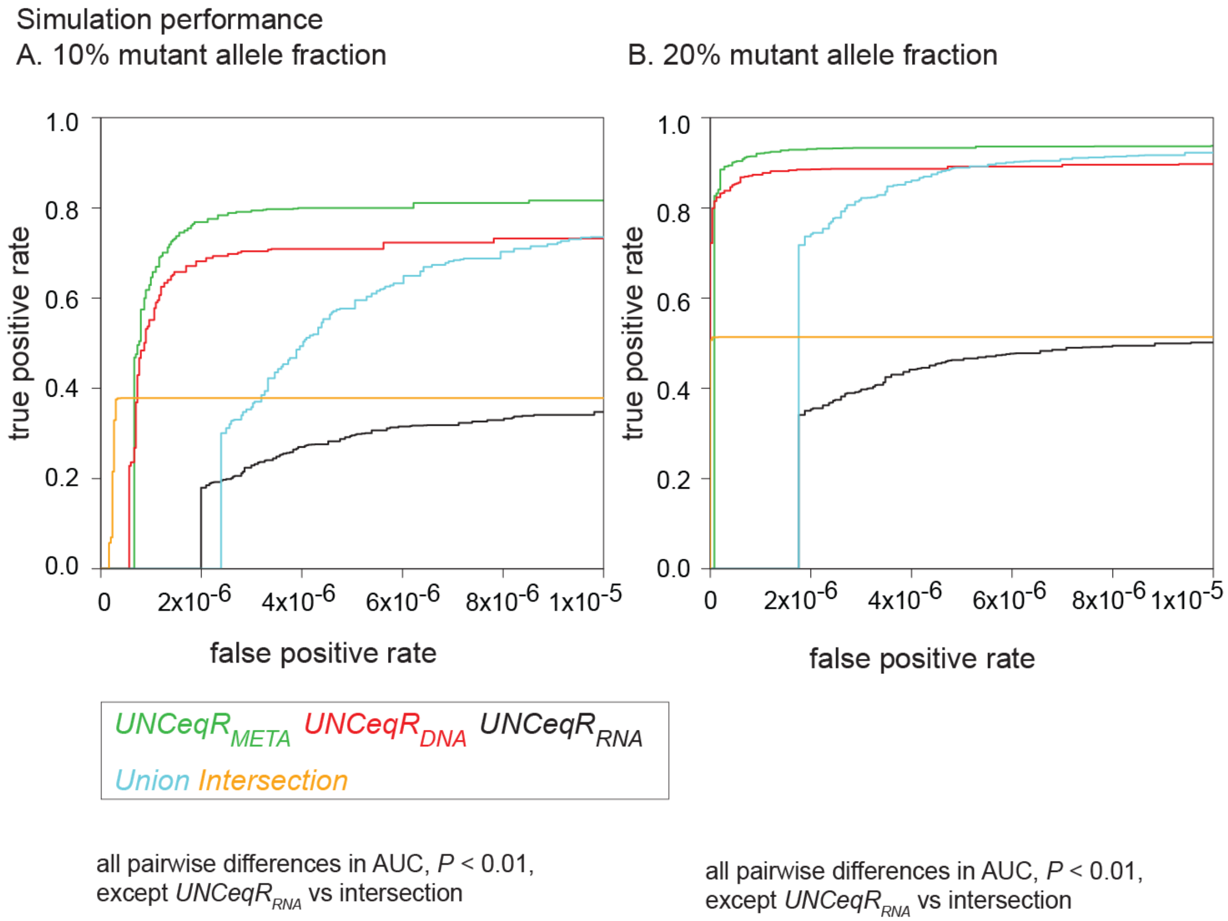

Figure S4: Extended validation analysis by whole genome sequencing

(A) Extended validation display corresponding to Figure 2, with additional models: *Union*, *Intersection*, and *Strelka_SNVmix.* As for Figure 2, the minimum tumor DNA WGS read count to positively confirm a mutation is 1. *Union* and *Intersection* were defined as in Figure S3. *Strelka_SNVmix* refers to the intersection of Strelka's DNA-WES ranked mutation predictions with SNVmix tumor RNA-seq mutation prediction irrespective of SNVmix. The union of SNVmix and Strelka predictions resulted in over 45,000 at the strongest predictive score even after filtering SNVmix germline RNA-seq calls and removing population and mapping artifacts. This large number is due to the inclusion of SNVmix. At the strongest predictive score, the validation rate (number of TP over all calls) of the SNVmix calls was 5% (calculated from a random subset of the data for feasibility), giving an approximate best performance of 42,300 false positives and 2,700 true positives (the Strelka top rank calls are very small in number and shift these estimates minimally). This point, which is the best case for the Strelka-SNVmix union model, represents a tremendously larger false positive rate than all other models. SNVmix does not include advanced RNA data filtering, e.g. as described in [28], which is part of *UNCeqR*, potentially explaining this large false positive rate of SNVmix.

(B) Validation by DNA whole genome sequencing requiring a minimum of 2 reads to confirm a mutation prediction. The ranking and shapes of the curves were maintained from (A) and the differences continued to be statistically significant.

A. Validation by DNA whole genome sequencing with ≥ 1 tumor variant reads

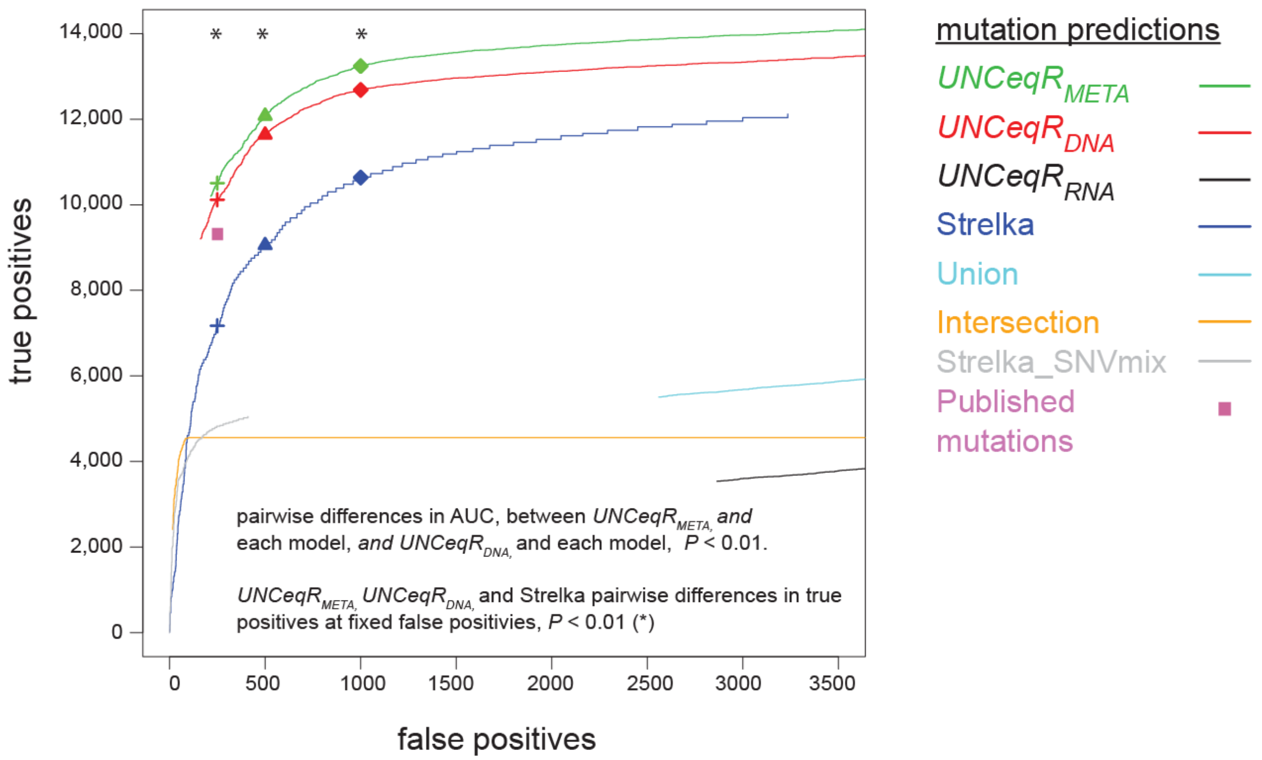

B. Validation by DNA whole genome sequencing with ≥ 2 tumor variant reads

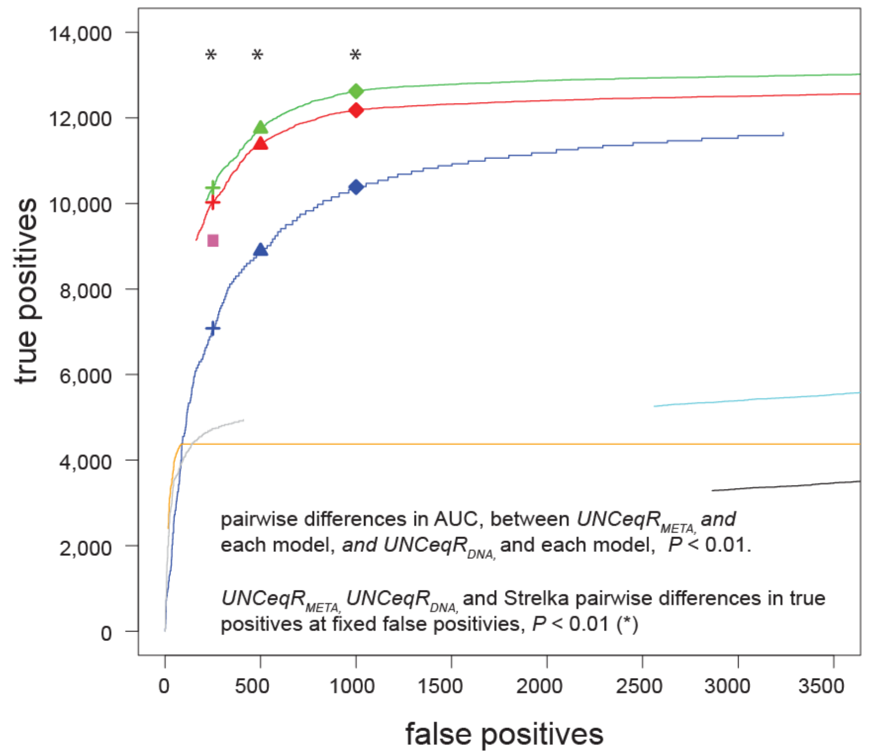

Figure S5: Review of previously validated mutations.

Proportions of 6,922 mutations, published as validated from [4] and [6], that were detected by $UNCeqR_{META}$ over different significance thresholds.

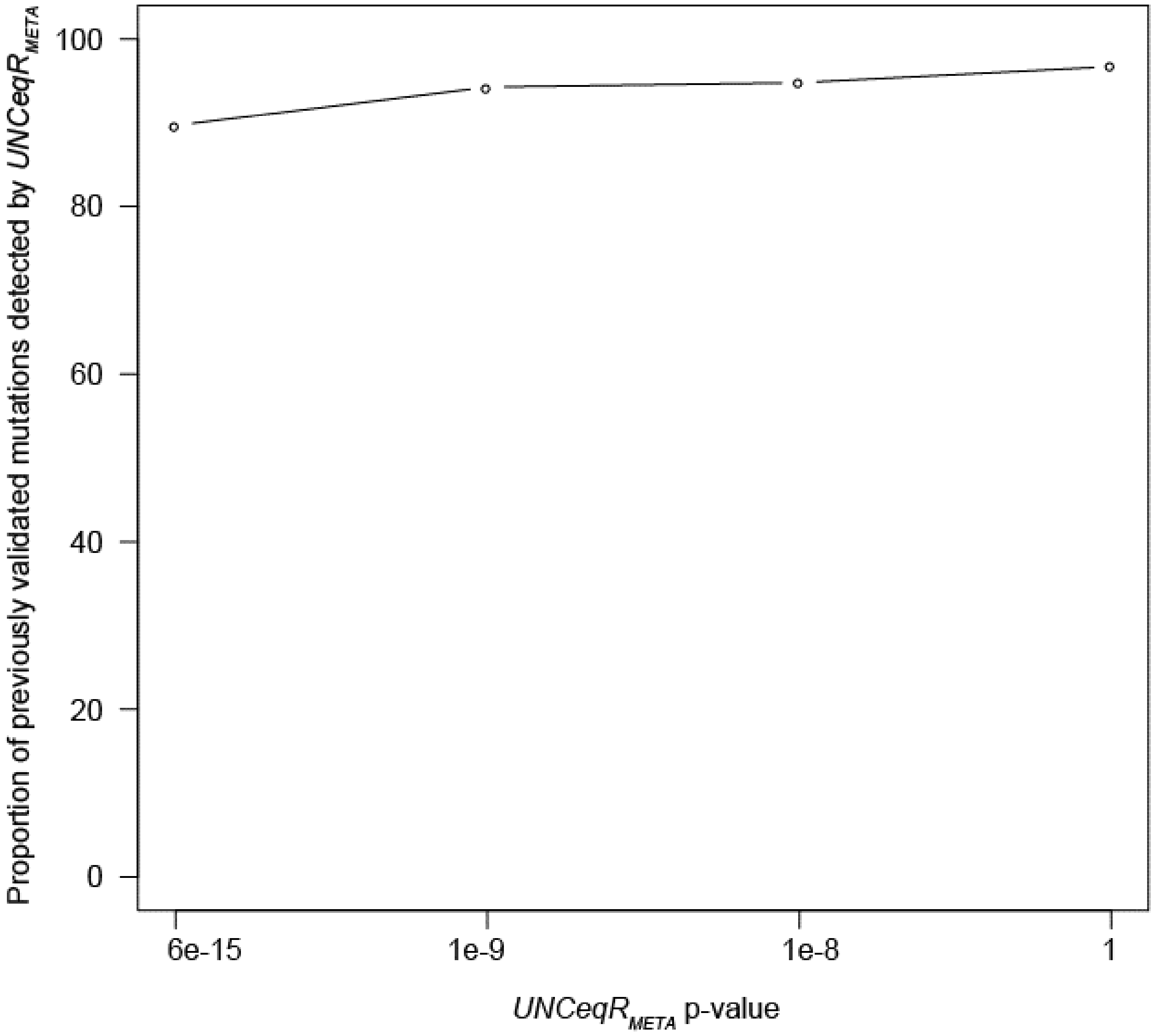

Figure S6: Allele fractions of somatic mutations and germline polymorphisms in breast cancer.

Mutant allele fractions displayed from expressed mutations of breast triplet cohort (A). Variant allele fractions displayed from expressed germline polymorphisms from germline sequencing of breast cancer quadruplet cohort (B).

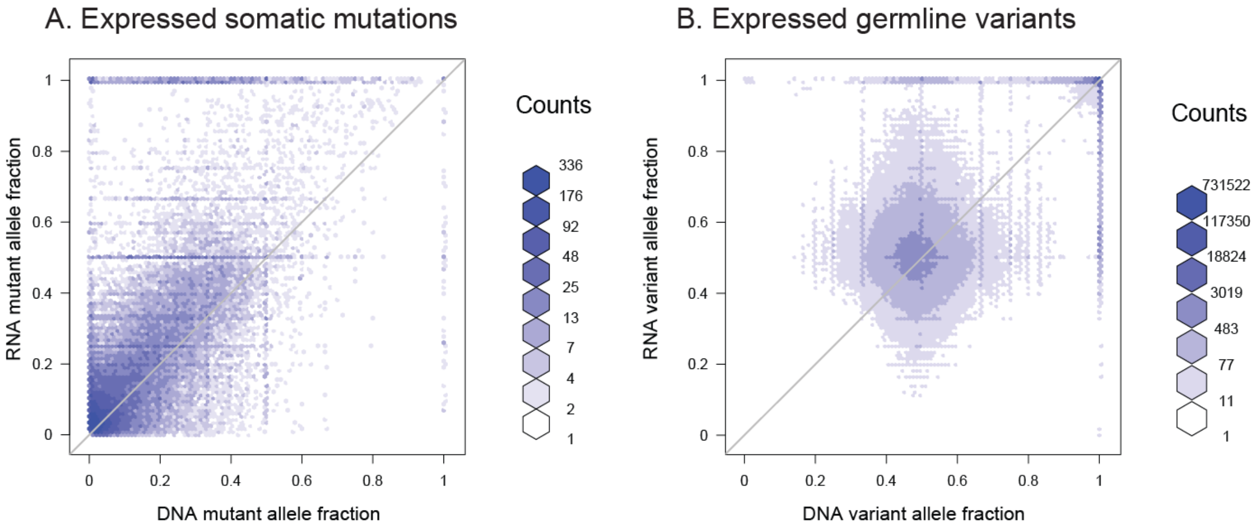

Fig S7: Copy number analysis

UNCeqR$_{META}$ mutations were subset into sets, whether the RNA mutation allele fraction (MAF) is significantly greater than DNA MAF, DNA MAF is significantly greater than RNA MAF, or no significant difference in MAF. Each mutation was assigned a total DNA copy number from TCGA SNP array analysis. The proportions of these mutation sets having different total DNA copy number were displayed as barplots. Over all mutations, mutations with greater MAF in RNA *vs* DNA had a small enrichment for copy number loss while mutations with greater MAF in DNA *vs* RNA had a small enrichment in copy number amplifications. Restricting to mutations in *TP53*, no significant association was found. *P* indicates chi-square test on copy number versus mutation set (DNA MAF > RNA MAF, or RNA MAF > DNA MAF).

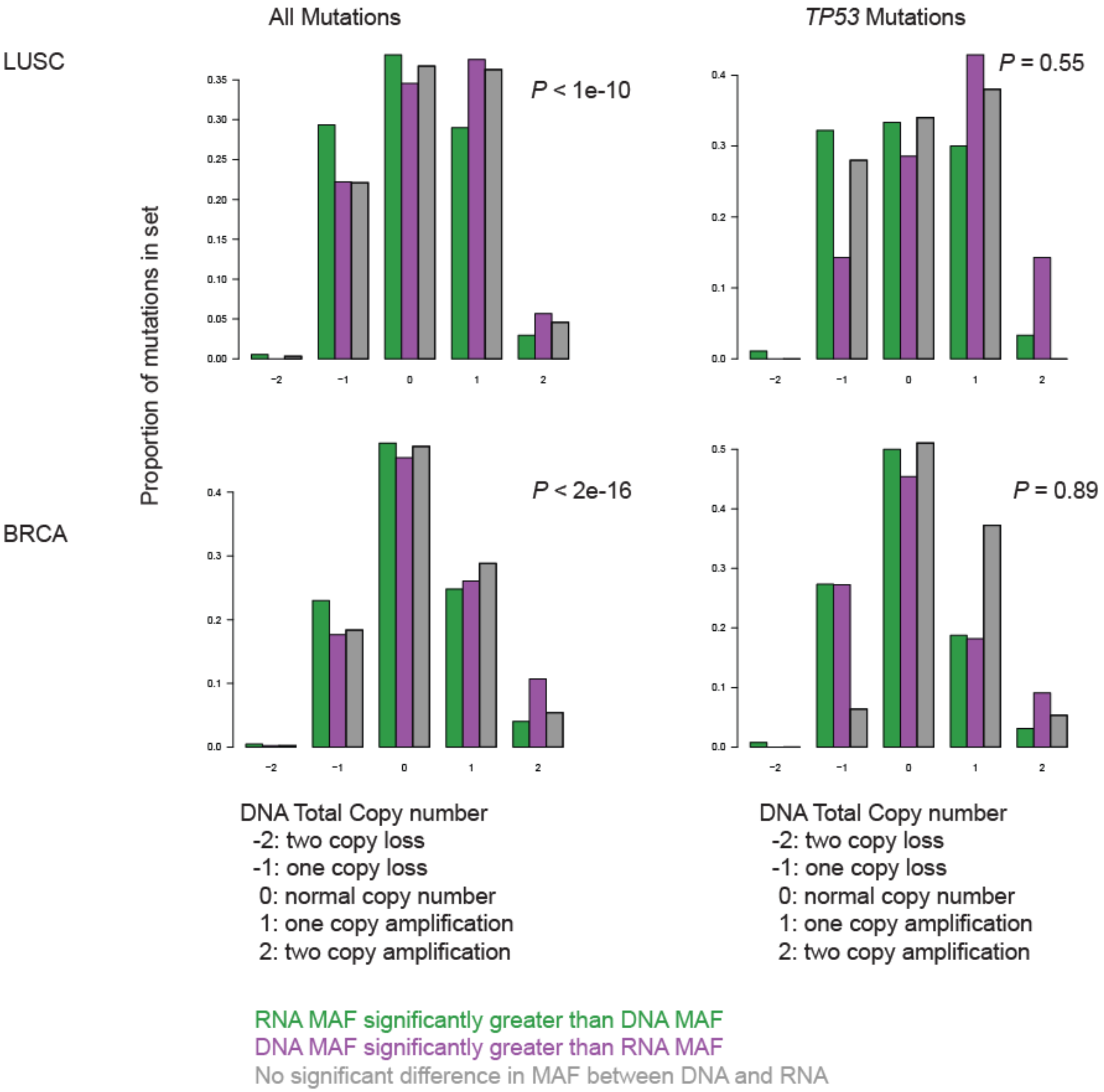

Fig S8: Breast cancer mutations detected in *GATA3.*

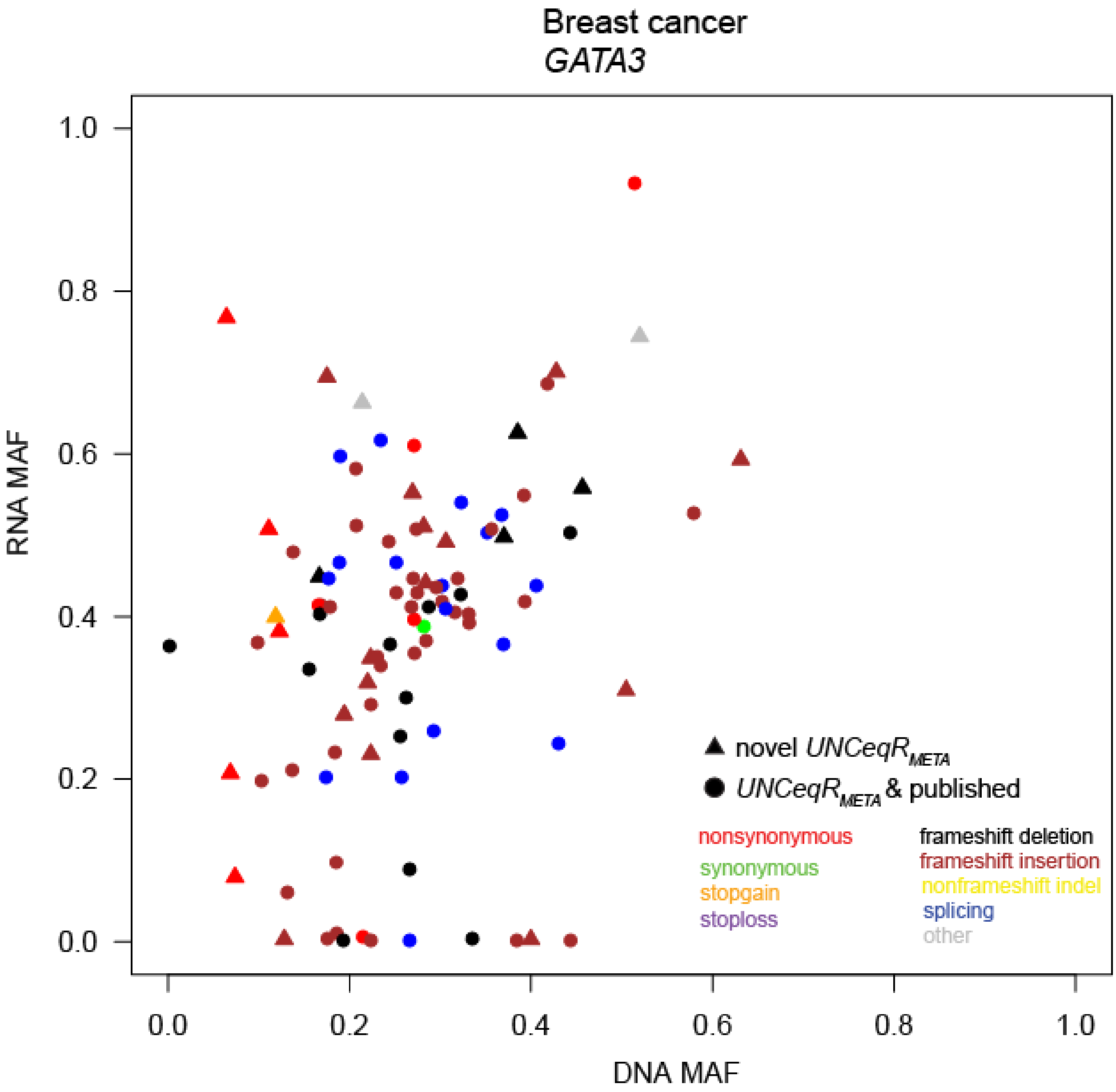

Table S1. Sequencing alignment identifiers.

Sequencing alignments (BAM files) analyzed in this study are listed by patient with corresponding identifiers. In some cases, multiple BAMs from separate sequencings were available for some specimen types such as two germline DNA-WES. The primary BAMs used in this study are labeled “modelComp”. All available BAMs were used when comparing to published profiles (Fig. 6) and these records are labeled “tcgaComp”.
